# Phytochemistry, Mechanisms, and Preclinical Studies of Echinacea Extracts in Modulating Immune Responses to Bacterial and Viral Infections: A Comprehensive Review

**DOI:** 10.3390/antibiotics13100947

**Published:** 2024-10-09

**Authors:** Fatemeh Ahmadi

**Affiliations:** 1 UWA School of Agriculture and Environment, The University of Western Australia, Perth 6009, Australia; fatemeh.ahmadi@uwa.edu.au; 2 Tasmanian Institute of Agriculture, University of Tasmania, Hobart 7001, Australia

**Keywords:** alkamides, caffeic acid derivatives, COVID-19, immune system, medicinal plant, phytochemistry

## Abstract

Background: Echinacea species, particularly *Echinacea purpurea*, *Echinacea angustifolia*, and *Echinacea pallida*, are renowned for their immunomodulatory, antibacterial, and antiviral properties. Objectives: This review explores the mechanisms by which echinacea herbal extracts modulate immune responses, focusing on their effects on both innate and adaptive immunity in bacterial and viral infections. Results: Key bioactive compounds, such as alkamides, caffeic acid derivatives, flavonoids, and polysaccharides, contribute to these effects. These compounds enhance immune cell activity, including macrophages and natural killer cells, stimulating cytokine production and phagocytosis. The antibacterial activity of echinacea against respiratory pathogens (*Streptococcus pneumoniae*, *Haemophilus influenzae*, *Legionella pneumophila*) and skin pathogens (*Staphylococcus aureus*, *Propionibacterium acnes*) is reviewed, as well as its antiviral efficacy against viruses like herpes simplex, influenza, and rhinovirus. Echinacea’s potential as a complementary treatment alongside conventional antibiotics and antivirals is discussed, particularly in the context of antibiotic resistance and emerging viral threats. Conclusions: Challenges associated with variability in phytochemical content and the need for standardized extraction processes are also addressed. This review provides a comprehensive overview of echinacea’s therapeutic potential and outlines future directions for research, including clinical trials and dosage optimization.

## 1. Introduction

Herbal and botanical products have been utilized for disease prevention and treatment for thousands of years. Many Native Indian tribes and Asian cultures across the globe are recognized for their significant role in advancing botanical medicine [1]. It is estimated that more than 30,000 herbs and botanicals have been explored for medicinal purposes, though fewer than 300 are actively incorporated into Western medicine today [2]. The genus Echinacea (Asteraceae) includes a small group of hardies, herbaceous perennial species indigenous to parts of North America [3]. *Echinacea purpurea*, derived from a flowering plant, is a popular herbal medicine. This plant is native to the United States, particularly in regions east of the Rocky Mountains, and the Atlantic drainage area, including the Great Plains, and extends to the central U.S. and nearby areas of Canada [4]. According to the Royal Botanic Kew Gardens’ “Plants of the World Online”, native Echinacea species range from Western Europe to Southeastern Asia [5]. There are at least nine species of echinacea, each potentially varying in medicinal properties. Three species—*Echinacea angustifolia* (DC.) Hell., *Echinacea pallida* (Nutt.) Nutt., and *Echinacea purpurea* (L.) Moench—are commonly used for therapeutic purposes [6]. Some vernacular names for echinacea species include black Sampson, coneflower, pale purple coneflower (*E. pallida*), purple coneflower (*E. purpurea*, *E. angustifolia*), narrow-leaf purple coneflower (*E. angustifolia*), and Kansas snakeroot (*E. angustifolia*) [7]. The medicinal parts include the fresh or dried roots and rhizomes of all three species, with *E. purpurea* also using its fresh or dried flowering tops and fresh-pressed juice [8]. Commercial echinacea products, which may contain one or more of these raw materials from different regions, are available in various forms such as tinctures, tablets, teas, capsules, and parenteral preparations [9]. Echinacea has a long-standing history in medicine for treating diverse conditions, including infections such as syphilis and septic wounds, and has been historically utilized as an “anti-toxin” for snakebites and blood poisoning [10].

Historically, echinacea was regarded as an “anti-infective” agent, commonly used to address bacterial and viral infections, mild septicemia, furunculosis (frequent painful skin nodules), and a range of skin conditions, such as boils, carbuncles, and abscesses [11]. It was also traditionally employed in the treatment of nasopharyngeal catarrh, pyorrhoea (periodontitis), and tonsillitis, as well as a supportive remedy for flu-like symptoms, recurring respiratory tract infections, and urinary tract infections. Externally, it was applied to poorly healing superficial wounds [12]. In recent efforts to substantiate these traditional uses, numerous studies have investigated the effects of standardized *E. purpurea* formulations on pathogens, inflammatory processes, and gene expression in both infected and healthy human cells and animal models [13]. Today, echinacea is primarily recognized as an over-the-counter herbal remedy for colds and flu and is also used for pain, inflammation, migraines, and other health conditions. However, in some regions, echinacea is regulated as a licensed medicinal product and is even prescribed by doctors, although such prescriptions are not always required [14]. The belief that echinacea should be avoided in autoimmune diseases assumes that boosting any aspect of immune function could be harmful [15]. However, the immune system is highly complex, and substances primarily enhancing phagocytic activity may be safe or beneficial in autoimmune disorders [16,17]. A case study on the long-term use of echinacea in chronic lymphocytic leukemia found no negative effects and underscored its benefits for immune function [18].

**Figure 1:**
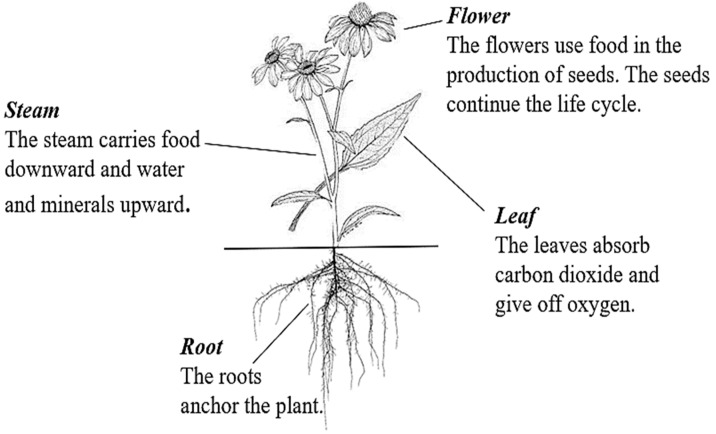
Botanical illustration of *E. purpurea*, depicting the aerial and root structures (Figure drawn by F. Ahmadi).

Echinacea species, now commonly recognized as immune stimulants, were historically used by North American Indigenous Peoples to treat throat infections, wounds, and pain. In the past, echinacea was also employed in eclectic medicine to treat septic conditions [19]. Ongoing research continues to investigate its pharmacological properties and potential therapeutic applications, including anti-inflammatory, analgesic, anxiolytic, and antimicrobial effects [20]. Echinacea is generally considered safe, with severe side effects being rare. Most users experience few adverse effects, with mild reactions such as gastrointestinal discomfort or skin irritation occurring infrequently [21]. However, like any herbal supplement, there is a potential for allergic reactions, particularly in individuals allergic to members of the Asteraceae family, which includes ragweed, chrysanthemums, marigolds, and daisies [13]. The morphology of the echinacea is depicted in Figure 1.

## 2. Methodology

Multiple academic databases were utilized to conduct a comprehensive literature search, including Web of Knowledge, Scopus, ScienceDirect, Google Scholar, Web of Science Core Collection by Clarivate Analytics, and ResearchGate. The search focused on publications from 1989 to 2024. Several keywords were employed to obtain a wide range of search results. These keywords encompassed various aspects related to echinacea species, phytochemistry, medicinal plants, echinacea biology, biochemistry, bioactive compounds, antibacterial properties, antivirus, viral infections, immune response, traditional medicine, biological models, and antibacterial mechanisms. In addition to the initial search, the references cited within the obtained publications were collected to ensure a comprehensive literature review. The chosen keywords were combined using operators like “OR” and “AND” to refine the search and obtain more precise results. Including quotation marks around specific terms, such as “echinacea”, ensured accurate retrieval of relevant records. All the keywords were used in all six databases to maximize search coverage and gather a comprehensive collection of literature on the subject. The keywords and topics that are trending in each specific review section were identified. To address the research question and achieve the review objective, a thorough search for peer-reviewed studies was conducted specifically focused on echinacea’s medicinal properties and mechanisms. The search was primarily centered on journal articles, excluding grey literature such as books, book chapters, and conference papers, except in rare cases where they provided valuable insights. The author screened and evaluated titles and abstracts from over 255 articles, employing a rigorous selection process to identify relevant papers. A key criterion for inclusion was the presence of quantitative information within the study. Specifically targeted studies addressed the following topics: (i) echinacea species phytochemistry, physiology, and medicinal properties; and (ii) the mechanisms of echinacea bioactive compounds on bacterial and viral infections. After comparing and analyzing the remaining articles, we categorized them based on relevant keywords. Additionally, the author summarized the conducted research, critically evaluated the content of over 192 studies, extracted essential features, and identified key challenges that warrant further investigation. By following this rigorous methodology, the author aimed to provide a comprehensive review that synthesizes the current state of knowledge, highlights significant findings, and identifies areas that require additional research attention within the context of echinacea phytochemistry and medicinal properties.

## 3. Phytochemistry

The composition of echinacea varies between species and their different plant parts [12]. It is commonly believed that no single compound or group of compounds is solely responsible for the effects of echinacea [22]. Rather, several classes of compounds—including alkamides, caffeic acid derivatives, polysaccharides, and alkenes (such as polyenes)—are thought to collectively contribute to its activity [23]. Below is a summary of the key constituents found in various echinacea species, compiled from several references, along with the structural formulas of some of these components [24].

### 3.1. Alkamides

**Figure 2:**
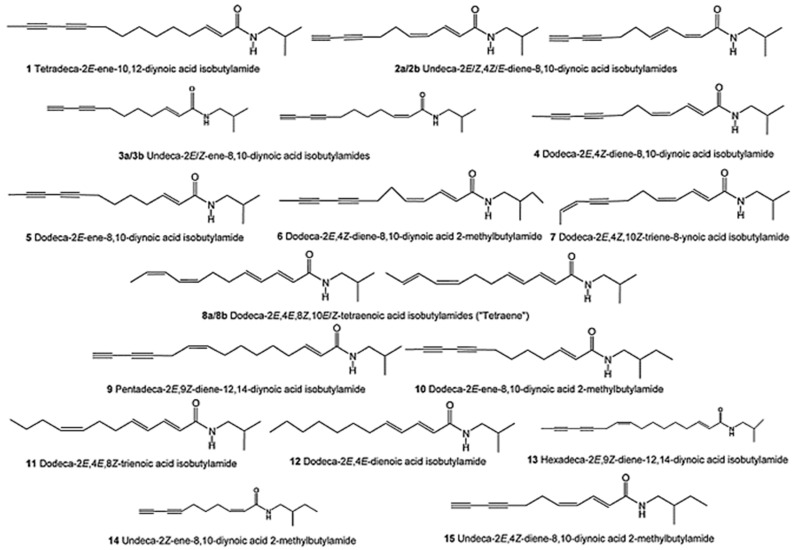
Structure of the main echinacea alkamides (Figure drawn by F. Ahmadi).

Alkamides are plant-derived, lipophilic compounds characterized by a combination of various amines and aliphatic acids connected via an amine linkage [25]. These amine residues, which can be either aromatic or aliphatic, are produced through the decarboxylation of amino acids [26]. The main alkamides found in different echinacea species are depicted in Figure 2, including the isomeric dodeca-2E, 4E, 8Z, and 10E/Z-tetraenoic acid isobutylamide (8a/8b) (Figure 2). Isobutylamine originates from the amino acid valine and is the most common amine present in alkamides [27]. Other frequent amine residues include piperidine—derived from lysine, pyrrolidine—derived from ornithine, N-2-methylbutyl—whose precursor is isoleucine, and N-phenethyl—derived from phenylalanine [28]. A characteristic feature of echinacea alkamides is the attachment of isobutyl or 2-methylbutyl amines to an unsaturated fatty acid chain, which contains one or more double or triple bonds [29]. However, two alkamides isolated from hydroalcoholic extracts of *E. purpurea* roots possess a hydroxyl group and a carboxylic acid at the terminal end of the fatty acid chain [30]. Alkamide concentrations vary significantly between the roots, stems, and flowers of *E. purpurea*, with higher levels of dodeca-2,4-diene-8,10-diyne alkamides in the roots, while dodecatetraene alkamides and nonadeca-2,4-diene-8,10-diynes are more prevalent in the stems [31]. A distribution study found that the root bark and secondary roots of *E. angustifolia* lacked alkamides. High-quality *E. purpurea* root material contains up to 6 mg/g of alkamides [32].

### 3.2. Flavonoids

**Figure 3:**
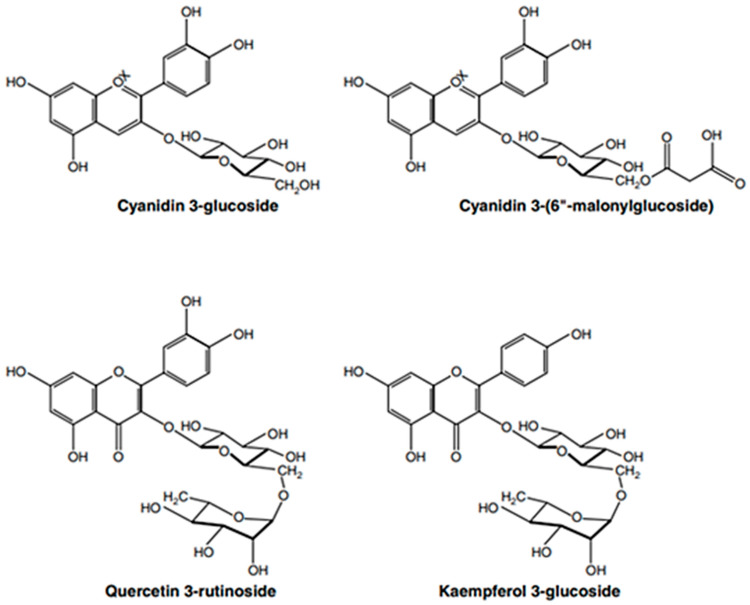
Flavonoids found in echinacea species (Figure drawn by F. Ahmadi).

There are only three documented reports of flavonoids in echinacea species. Previous research [33] identified the main anthocyanin pigments in the flowers of *E. purpurea* and *E. pallida* as cyanidin 3-glucoside and cyanidin 3-(6″-malonylglucoside) (Figure 3). Earlier studies [34] also produced anthocyanin-containing callus and suspension cultures from the stem of *E. purpurea*. From these suspension cultures, three anthocyanins were isolated: cyanidin 3-glucoside and two additional acylated cyanidin glycosides that were not fully characterized [35]. The only other report comes from unpublished data in a doctoral thesis, which noted trace amounts of quercetin and kaempferol glycosides, including the 3-rutinosides (rhamnosyl (1→6) glucosides, 56, 57), in the aerial parts of *E. purpurea* [36].

### 3.3. Hydrocarbons

Hydrocarbons are key root constituents of *E. pallida*, with approximately 11 derivatives identified, primarily ketoalkenes and ketoalkynes (polyacetylenes). The major compounds include the ketoalkenes—(Z)-pentadec-8-en-2-one, (8Z,11Z)-pentadeca-8,11-dien-2-one [37], (8Z,11Z,13E)-pentadeca-8,11,13-trien-2-one, and (8Z,11E,13Z)-pentadeca-8,11,13-trien-2-one [38]—and the ketoalkenynes—(8Z,13Z)-pentadeca-8,13-dien-11-yn-2-one, (Z)-tetradeca-8-diene-11,13-diyn-2-one [39], and (Z)-pentadeca-8-ene-11,13-diyn-2-one [40]. Additionally, two notable alkenes, pentadec-1-ene [41] and (Z)-pentadeca-1,8-diene [42], have been detected in the roots of *E. angustifolia* [43]. In contrast, the only hydrocarbon reported from *E. purpurea* roots is dodeca-2,4-dien-1-yl isovalerate, which is also found in the roots of *E. angustifolia* [44]. The absence of polyacetylenes in the roots of *E. angustifolia* and *E. purpurea* serves as a distinguishing factor between these species and *E. pallida*. Research has demonstrated that commercially available *E. pallida* root preparations are often contaminated with *E. angustifolia* [45]. This suggests that some early reports of hydrocarbons in *E. angustifolia* roots may have been due to misidentification or contamination with *E. pallida*. The hydrocarbons identified in the rhizomes of echinacea species are listed in Table 1.

### 3.4. Polysaccharides

Two immunostimulatory polysaccharides, PS I and PS II, have been isolated from the aerial parts of *E. purpurea*. PS I is identified as 4-O-methyl-glucuronoarabinoxylan with an average molecular weight of 35,000, while PS II is an acidic arabinorhamnogalactan with a molecular weight of 50,000. Both of these polysaccharides exhibited significant activity in various in vitro and in vivo immunological tests [47]. A crude polysaccharide extract from the roots of *E. purpurea* has not been fully analyzed, though it seems to have a similar composition to that of the aerial parts [48]. Additionally, from cell cultures of *E. purpurea*, three homogeneous polysaccharides were obtained: two neutral fucogalactoxyloglucans, with molecular weights of 10,000 and 25,000, and an acidic arabinogalactan, with a molecular weight of 75,000 [49]. The fucogalactoxyloglucan with a molecular weight of 25,000 was found to enhance phagocytosis in both in vitro and in vivo assays, while the arabinogalactan specifically triggered macrophages to secrete tumor necrosis factor (TNF) [50,51]. The acidic arabinorhamnogalactan from *E. purpurea* is now produced biotechnologically on an industrial scale and is being considered for clinical trials [52]. Previous research classified this polysaccharide as a type II arabinogalactan, characterized by a (1→3)-linked β-D-galactan backbone, which is likely attached to rhamnogalactan and arabinan chains [53,54]. The polysaccharides produced through tissue culture differ structurally from those found in the aerial parts, as they are components of the primary cell walls in cultured cells. As a result, the polysaccharides derived from the aerial parts of *E. purpurea* show little resemblance to those produced by tissue cultures [55].

### 3.5. Caffeic Acid Derivatives (CADs)

**Figure 4:**
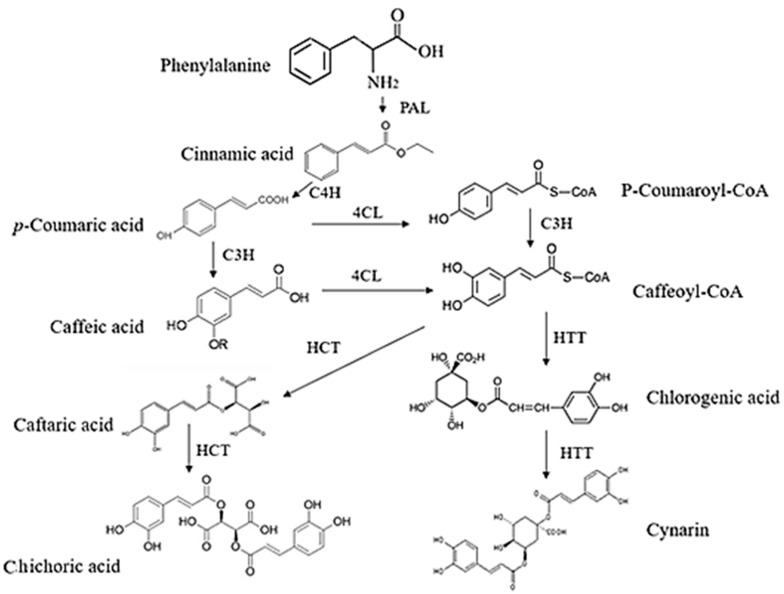
A possible biosynthesis pathway for chicoric acid and caffeic acid is derived via the phenylpropanoid pathway in echinacea species. PAL: phenylalanine ammonia-lyase, C4H: cinnamate-4-hydroxylase, C3H: coumarate 3-hydroxylase, 4CL: 4-coumarate-CoA ligase, HCT: shikimate o-hydroxycinnamoyl transferase (Figure drawn by F. Ahmadi).

Another important group of phytochemicals contributing to echinacea’s pharmacological effects is CADs (Figure 4). Unlike alkylamides, which are more limited in their distribution across taxa, phenolic metabolites like CADs are widely distributed among plants. Although echinacea has a unique collection of CADs, these compounds are found across many plant families [56,57]. Two of the most extensively studied CADs in *E. purpurea* are caftaric acid and chicoric acid, as they represent the dominant polyphenols in this species [58]. In addition to their role in plant defenses, such as deterring herbivores and providing interspecies protection, CADs are associated with echinacea’s immunostimulatory and antioxidant properties [59]. However, oral administration studies indicate that these compounds have poor bioavailability, raising questions about their effectiveness in humans [60]. Chicoric acid is particularly abundant in *E. purpurea*, making up as much as 20% of total CADs in the roots, while the flowers, stems, and leaves contain approximately 35%, 10%, and 35%, respectively [61]. For natural health products, this distribution suggests that products should prioritize flowers and leaves, which contain higher concentrations of CADs. Some natural health products reflect this, whereas others focus on alkylamides, which are mainly concentrated in the roots and found in smaller amounts in the aerial parts [62].

## 4. Antibacterial Activities of Echinacea Species

There are numerous reports detailing the traditional use of echinacea species in treating bacterial infections [63]. Three species in particular, *E. purpurea*, *E. angustifolia*, and *E. pallida*, have historically been used as antibacterial remedies. Research [64] has also discussed the application of *E. purpurea* in Brazil, where a leaf infusion is applied topically to infected areas [65]. Several studies have since been conducted to validate the antibacterial properties traditionally attributed to echinacea species and their preparations [66]. One such study investigated the antibacterial activity of a fermented extract of *E. purpurea* (5% *w*/*v*, fermented with Lactobacillus plantarum) [67]. Using disc diffusion and broth microdilution assays, the extract was tested on a range of bacterial strains, including *E. coli*, *Enterobacter aerogenes*, *Enterococcus durans*, *Yersinia enterocolitica*, *Weissella confusa*, *Leuconostoc lactis*, *Propionibacterium jensenii*, *Lactobacillus sakei*, and *Bacillus megaterium*. The results demonstrated that the fermented extract inhibited the growth of most of the strains tested, with the greatest inhibition seen in *B. megaterium* and *L. lactis*, where inhibition halos measured over 3.5 mm and between 2.5–3.5 mm, respectively. No inhibitory effect was observed for *L. sakei* [68].

A previous study [69] investigated the bactericidal effects of a 65% ethanol extract from freshly harvested aerial parts and roots of *E. purpurea* at concentrations of 40 and 120 mg of dry mass/mL. The microdilution method, using both light and dark assays, was applied to test the extract against strains responsible for respiratory infections, including *L. pneumophila*, *Streptococcus pyogenes*, *Mycobacterium smegmatis*, and *H. influenzae*. Results showed that *S. pyogenes*, *H. influenzae*, and *L. pneumophila* were sensitive to the higher concentration of the extract. Another study [70], employing agar diffusion tests, assessed the antibacterial activity of hydroethanol extracts (prepared via conventional and ultrasonic extraction) from the aerial parts of *E. purpurea* (20 mg/mL). The extracts were tested against standard strains such as *E. coli*, *P. aeruginosa*, *Bacillus subtilis*, and *S. aureus*. The conventional extraction method produced larger inhibition zones for all strains, with *E. coli* (11.2 ± 0.2 mm) and *B. subtilis* (10.9 ± 0.1 mm) showing the most significant inhibition. In another study [71], the antibacterial activity of *E. angustifolia* extracts was evaluated using solvents of increasing polarity (petroleum ether, methanol, and water) at concentrations of 0.5, 1.5, and 1.5 mg/mL, respectively, against *B. subtilis*, *S. aureus*, *E. coli*, and *Staphylococcus epidermidis*. The study did not specify the plant parts used for extraction, and the aqueous extracts were less effective than those prepared with other solvents.

Respiratory infections can be caused by various pathogenic bacteria, including *Streptococcus pneumoniae*, *H. influenzae*, *Moraxella catarrhalis*, *S. pyogenes*, *Bordetella pertussis*, *Mycoplasma pneumoniae*, *S. aureus*, *P. aeruginosa*, and *Burkholderia cepacia* [72], among others. In this context, plants from the echinacea genus have shown promising antibacterial activity against microorganisms involved in these infections, as demonstrated by previous studies [73]. Additionally, *E. purpurea* has been found to inhibit *L. pneumophila*, the bacterium responsible for pneumonia [74]. However, the results for *S. aureus* have been inconsistent, possibly due to variations in the echinacea species used, plant parts, extraction methods, and thus their chemical compositions. Earlier research [75] suggested that neither alkylamides nor polysaccharides alone were responsible for the reported bactericidal effects. Furthermore, no clear link was found between bactericidal activity and caffeoyl derivatives [76]. Conversely, other studies [77] have correlated antimicrobial activity with total phenolic and flavonoid content.

Several bacteria, including *P. acnes*, *S. aureus*, *S. pyogenes*, *P. aeruginosa*, *Pasteurella multocida*, *Capnocytophaga canimorsus*, *Bartonella* spp., *Klebsiella rhinoscleromatis*, and *Vibrio vulnificus*, are key pathogens involved in skin infections [78]. Studies have shown that echinacea plants possess active compounds with antimicrobial properties that target these skin pathogens, supporting their traditional use in ethnomedicine for treating skin infections [79]. Additionally, recent research has demonstrated the potential of *E. purpurea* in addressing throat and mouth conditions (Oto-rhino-laryngological, OTO). One study [80] revealed that extracts from *E. angustifolia* and its primary alkylamide isomers, dodeca-2E, 4E, 8Z, and 10Z/E-octadecenoic acid (alkylamide 8/9, which is also found in *E. purpurea*), inhibited the growth of *Candida albicans*, a leading cause of fungal throat infections [81]. These findings align with ethnobotanical evidence supporting the use of echinacea for OTO-related conditions. Moreover, echinacea has been reported to inhibit microorganisms responsible for respiratory infections, including *Legionella pneumophila*, *Streptococcus pyogenes*, and *Mycobacterium smegmatis* [82]. However, despite being a well-studied respiratory pathogen, *Pseudomonas* has not been thoroughly investigated concerning echinacea treatments [83].

Purple coneflower (*E. purpurea*) is known to counteract proinflammatory cytokine stimulation, regardless of the bacterial or viral source of infection [84]. Several studies have examined the effects of *E. purpurea* on the activation of lipopolysaccharide, an inflammatory mediator typically produced by *E. coli*, in various human cell cultures and animal models, as well as research involving live bacteria [85]. While these models do not always fully replicate live bacterial infections, they provide useful insights for testing potential anti-inflammatory agents. The findings suggest that *E. purpurea* acts as a general anti-inflammatory agent, capable of reducing several symptoms associated with respiratory infections [86]. Extracts obtained from the aerial parts of *E. purpurea* have shown stronger antibacterial and antioxidant properties compared to those derived via ultrasonic extraction methods [87]. Currently, antibiotics and vaccines are the main treatments for bacterial infections, used both therapeutically and prophylactically. However, managing respiratory infections remains a challenge due to the variety of microorganisms involved. In this context, the use of phytopreparations could offer a promising alternative [88]. To better understand the synergistic interactions between echinacea extracts and antibiotics, further research is necessary to elucidate the mechanisms of their antimicrobial effects and identify potential pathways that could be targeted [83].

## 5. Mechanism of Antibacterial Activity of Polyphenols

Echinacea species exhibit antimicrobial activity through several distinct mechanisms, which together enhance their effectiveness against a wide range of pathogens [89]. A key mechanism is the disruption of microbial cell membranes, primarily due to the hydrophobic compounds like alkamides present in echinacea [90]. These compounds can integrate into the lipid bilayers of microbial membranes, leading to structural instability and increased permeability, ultimately causing cell lysis and death [90]. Another important mechanism is the inhibition of microbial enzymes essential for survival and replication. Compounds such as caffeic acid derivatives inhibit enzymes responsible for processes like cell wall synthesis and nucleic acid production, thereby hindering microbial growth and reproduction [91]. Additionally, echinacea has immunomodulatory effects, stimulating immune cells like macrophages and natural killer cells, enhancing phagocytosis, and increasing cytokine production, which helps coordinate the body’s immune response to infections [92]. Furthermore, echinacea extracts have been shown to prevent biofilm formation—protective layers bacteria form to evade immune responses and resist antibiotics—making bacteria more vulnerable to immune defenses and antimicrobial treatments [93]. The antioxidant properties of echinacea also contribute by neutralizing free radicals generated during infections, reducing oxidative stress, and protecting host tissues from damage, which further supports its antimicrobial action [89]. Together, these multiple mechanisms position echinacea as a versatile antimicrobial agent, capable of combating a variety of pathogens through both direct antimicrobial activity and indirect immune system modulation [90].

### 5.1. Reactions with Proteins

The antibacterial activity of flavonoids may be attributed to their ability to form complexes with proteins through nonspecific interactions, such as hydrogen bonding and hydrophobic forces, as well as through covalent bond formation [94]. The binding of polyphenols to proteins results in the formation of soluble or insoluble complexes, which affects the functions of both polyphenols and proteins [95]. This interaction can cause certain amino acids in proteins to be blocked or lead to conformational changes, altering the protein’s structure, solubility, hydrophobicity, thermal stability, and isoelectric point [96]. As a result, protein–phenolic complexation can modify their physicochemical and biological properties, influencing the digestibility of food proteins, the activity of digestive enzymes, and nutrient availability [94]. It has been shown that polyphenols, such as condensed tannins, can inhibit several digestive enzymes, including α-glycosidase, α-amylase, lipase, pepsin, trypsin, and chymotrypsin, thereby modulating nutrient availability, and altering microbiota composition [95]. Additionally, polyphenols can bind to crucial bacterial proteins, such as adhesins and enzymes, and transport proteins in the bacterial cell envelope, inactivating them and exerting an antimicrobial effect. However, the complexation of polyphenols with proteins may also impact the bioaccessibility and activity of phenolic compounds [97].

Some flavones have shown activity against *E. coli* by forming complexes with extracellular and soluble proteins [94]. Flavonoids are also known to influence the activity of bacterial enzymes essential for cell survival, including those involved in synthesizing cell wall components, membrane fatty acids, or adenosine triphosphate (ATP). Fatty acid synthase II (FAS-II) is a critical enzyme in bacterial membrane fatty acid synthesis, catalyzing the elongation of fatty acid chains from 16–24 carbons produced de novo by FAS-I to longer chains of 36–48 carbons, as well as mycolic acids [97]. Flavonoids such as isoliquiritigenin, butein, fisetin, and4′-trihydroxy chalcone have been found to inhibit FAS-II, thus hindering the growth of Mycobacterium smegmatis [98].

### 5.2. Inhibition of Bacterial DNA Synthesis and Interaction with Nucleic Acids

Flavonoids derived from *Elaeagnus glabra* were tested for antibacterial effects against *Proteus vulgaris* and *Staphylococcus aureus* [99]. The presence of a free 3′, 4′, and 5′-trihydroxy B-ring, along with a free 3-OH group, was found to be necessary for antibacterial activity. In *P. vulgaris*, DNA synthesis was primarily inhibited by active flavonoids, while in *S. aureus*, RNA synthesis was inhibited [100]. Robinetin, myricetin, and (−)-epigallocatechin were identified as the most potent inhibitors of DNA synthesis. It is suggested that the B-ring of these flavonoids may interact with nucleic acid bases by forming hydrogen bonds or intercalating, leading to the inhibition of bacterial nucleic acid synthesis. A previous study [101] found that p-coumaric acid exhibited dual bactericidal actions: disrupting the bacterial cell membrane and binding to bacterial genomic DNA, which inhibited essential cellular functions and caused cell death [102]. In *S. aureus*, membrane depolarization and the inhibition of DNA, RNA, and protein synthesis were observed when treated with flavonoids from *Dorstenia* species, such as 6,8-diprenyleriodictyol, isobavachalcone, and 4-hydroxylonchocarpin. At higher concentrations, cell lysis occurred.

There is also evidence that flavonoids can inhibit bacterial type II topoisomerases, including DNA gyrase and topoisomerase II (also known as topoisomerase IV) [101]. These enzymes, which regulate DNA topology, are specific to prokaryotes, making them attractive targets for antibacterial drugs. DNA gyrase consists of two key subunits: DNA gyrase subunit A (GyrA), responsible for DNA cleavage and rejoining, and DNA gyrase subunit B (GyrB), which contains the ATP-binding site. Natural compounds like coumarins and cyclothialidines inhibit ATPase activity in DNA gyrase by blocking ATP binding to the GyrB subunit. Research [99] demonstrated that quercetin inhibits the supercoiling activity of bacterial gyrase and induces DNA cleavage, likely through interaction with DNA. Quercetin binds to the 24 kilodalton fragment of GyrB in *E. coli* with a dissociation constant of 15 µM, inhibiting ATPase activity by competing with ATP for the binding site. Quercetin’s binding site overlaps with the ATP-binding pocket and can be competitively displaced by either ATP or novobiocin [101]. The proposed mechanism suggests that quercetin inhibits gyrases by interacting with either the DNA or the ATP-binding site [101]. Other polyphenols, such as catechins, have also been found to inhibit bacterial DNA gyrase by binding to the ATP site on the GyrB subunit, with epigallocatechin gallate being the most active, followed by epicatechin gallate and epigallocatechin [102]. Flavonoids such as quercetin, apigenin, and 3,3′,4′,6,7-pentahydroxyflavone have also shown inhibitory activity against *E. coli* DNA gyrase [102].

### 5.3. Interaction with the Bacterial Cell Wall or Inhibition of Cell Wall Formation

Differences in antimicrobial activity between Gram-negative and Gram-positive bacteria may result from variations in their cell surface structures [103]. The main function of the bacterial cell wall is to provide shape, maintain cell integrity, and serve as an osmotic barrier. Gram-negative bacteria are known to be resistant to many antibacterial agents due to the hydrophilic nature of their outer membrane and the presence of enzymes in the periplasmic space, which can break down external molecules [104]. Additionally, the negatively charged lipopolysaccharide layer in the outer membrane acts as a protective shield against catechins. In contrast, Gram-positive bacteria, which lack an outer membrane, tend to be more vulnerable to the action of phenolic acids. This lack of an outer membrane allows phenolic acids to diffuse more easily through the cell wall and reach the intracellular environment [105]. It is suggested that one potential mechanism behind the antimicrobial action of phenolic acids involves hyperacidification at the plasma membrane interface, caused by the dissociation of these acids. This process disrupts the cell membrane potential, increases membrane permeability, and causes irreversible changes to the sodium-potassium ATPase pump, ultimately leading to cell death [104].

Flavones form complexes with cell wall components, which can inhibit bacterial adhesion and further microbial growth. Flavonoids with a C-7-modified naringenin core have been shown to inhibit bacterial enzymes, such as tyrosyl-tRNA synthetase. These compounds have also demonstrated inhibitory effects on the growth of *S. aureus*, *E. coli*, and *P. aeruginosa*. Baicalein, in particular, was found to be an effective bactericidal agent, and when combined with cefotaxime, it exhibited synergistic effects by inhibiting the expression of extended-spectrum β-lactamase beta-lactamase messenger RNA [105]. Another mechanism contributing to antibacterial activity is the inhibition of bacterial efflux pumps, which enhances the susceptibility of bacteria to existing antibiotics by causing membrane depolarization. Artonin, derived from *Morus mesozygia*, was effective against *S. aureus* by blocking efflux mechanisms and inducing membrane depolarization [103]. Furthermore, artonin was able to reverse multidrug resistance, lowering the minimum inhibitory concentrations of antibiotics and increasing their potency.

### 5.4. Alteration of Cytoplasmic Membrane Function

The bacterial cell membrane is essential for multiple vital functions, including osmoregulation, respiration, transport, biosynthesis, and the cross-linking of peptidoglycan and lipids [106]. Any disruption to its structure or function can result in metabolic failure and cell death, making membrane disruption a key mechanism in the antibacterial action of polyphenols. For example, catechins have been shown to damage bacterial membranes by binding to the lipid bilayer, inhibiting the synthesis of both intracellular and extracellular enzymes [107]. Apigenin was observed to cause dysfunction in fungal membranes, increasing permeability and triggering the release of small intracellular components such as ions and sugars, but not proteins [107]. Epicatechin-3-gallate and caffeic acid were found to target both the cell wall and cytoplasmic membrane of *P. aeruginosa*, leading to membrane destruction, increased permeability, and the enhanced entry of hydrophobic antibiotics. This process also caused the release of potassium ions and the leakage of nucleotides. Due to their partially lipophilic properties, phenolic acids can penetrate the cell membrane via passive diffusion, increasing permeability, reducing intracellular pH, and causing protein denaturation [108]. A methanol extract from Coriolus versicolor, rich in polyphenols, inhibited cell division in *S. aureus* by interfering with septum formation and causing the accumulation of peptidoglycan and teichoic acid precursors in the cytoplasm [107]. In *Salmonella enteritidis*, the extract damaged the cell envelope. Furthermore, at higher concentrations, catechins have been shown to induce the generation of reactive oxygen species, leading to oxidative stress, altered membrane permeability, and subsequent membrane damage.

Flavonoids and flavonol have been shown to destabilize membrane structures by disrupting and disorienting membrane lipids, leading to leakage from vesicles [106]. An inverse relationship was observed between the number of hydroxyl groups in flavonoids and their ability to induce leakage. Previous research [93] suggested that flavonoids lacking hydroxyl groups on their B rings were more effective at inhibiting microbial growth compared to those with –OH groups. On the other hand, previous research found that the presence of hydroxyl groups in the phenyl rings A and B generally did not affect the antibacterial activity level of flavones [107]. A notable increase in activity was observed for hydroxy derivatives of flavones, specifically against *S. aureus*. Interestingly, in contrast to other studies, the compounds tested showed greater effectiveness against Gram-negative bacteria such as *E. coli* and *P. aeruginosa* than against Gram-positive bacteria like *Enterococcus faecalis* and *Staphylococcus aureus* [108].

## 6. Antiviral Activities of Echinacea Species

In vitro studies have demonstrated that aqueous extracts of *E. purpurea* Moench are effective against both acyclovir-resistant and acyclovir-susceptible strains of HSV-1 and HSV-2 (herpes simplex virus) [109]. Specifically, HSV-1 was inhibited by chicoric acid, and a hexane extract derived from the plant’s roots [110]. Additionally, chicoric acid was shown to inhibit the integrase enzyme of human immunodeficiency virus type 1 (HIV-1) [111]. Mouse embryonic fibroblasts incubated with alcoholic root extract and plant juice for 24 h were resistant to infection by herpes, influenza A2, and the vesicular stomatitis virus [112]. Various influenza virus strains, including avian (H7N7 and H5N1), influenza A (H1N1 and H3N2), and the pandemic swine-origin H1N1, were significantly inhibited by direct contact with standard echinacea preparations [113]. Hemagglutination (HA) assays revealed that these preparations suppressed HA activity, suggesting they may block viral entry into cells [114]. In influenza A H1N1-infected mice, administration of a polysaccharide extract from the plant resulted in weight loss, although lung virus titers remained similar between treated and untreated animals [115]. However, treated mice exhibited reduced levels of keratinocyte chemoattractant, interleukin-10 (IL-10), and systemic interferon-gamma, indicating that *E. purpurea* may modulate cytokines to reduce the clinical symptoms of influenza [116]. Recent studies suggest that different plant components may positively impact influenza patients through various mechanisms [117]. To establish *E. purpurea* as a biological agent, further research—especially in vivo studies and assessments of potential toxicity—is needed [118]. Ongoing clinical trials related to SARS-CoV support the use of *E. purpurea* against coronaviruses. Due to structural similarities among coronaviruses, preparations of *E. purpurea* (L.) Moench could serve as a preventative treatment for all coronaviruses, although additional studies are required [119].

A randomized, double-blind, placebo-controlled clinical trial was conducted to assess the efficacy of *E. purpurea* in preventing infections caused by rhinovirus type 39 (RV-39) [120]. It was reported that 59% of the 48 participants who were infected and treated with pressed juice from the aerial parts of *E. purpurea* in a 22% alcohol base developed a cold, compared to 86% of the placebo group (*p* = 0.0883, according to Fisher’s exact test) [121]. Meanwhile, *E. pallida* var. *angustifolia* exhibited significant anti-rhinovirus activity. Root extracts of *E. pallida* var. *angustifolia* in ethanol 70%, hexane, and ethyl acetate demonstrated anti-RV activity, with MIC100 (the minimum concentration required to inactivate 100% of the virus) values of 62, 69, and 85 µg/mL, respectively. The study concluded that alkamide-rich hexane fractions were more effective against rhinovirus due to their lower MIC100 compared to ethyl acetate fractions, which contained fewer alkamides [122]. Treatment with echinaforce, a standardized preparation from freshly harvested *E. purpurea* including root extracts in a 65% *v*/*v* ethanol solution, showed a slight reduction in viral titers at the highest dose of 50 µg/mL in post-infection cell lines with HCoV-229E [123]. When exposed to echinaforce, HCoV-229E was irreversibly inactivated, with a 50% inhibitory concentration (IC50) of 3.2 µg/mL, as demonstrated through 3-(4,5-dimethylthiazol-2-yl)-2,5-diphenyltetrazolium bromide assays conducted on Huh-7, Vero, and Vero E6 cells [124]. Additionally, it is reported that [121] the efficacy of *E. purpurea* for the prevention of rhinovirus colds, further supporting its potential clinical applications.

The aqueous fraction of *E. purpurea* roots has been reported to show potent antiviral activity against the influenza virus, whereas the ethyl acetate fraction from *E. angustifolia* root extracts demonstrated moderate activity against the herpes simplex virus, influenza, and rhinovirus [125]. In contrast, extracts from *E. pallida* roots exhibited no antiviral activity [126]. The minimum concentration of *E. purpurea* needed to inactivate 100% of the virus (MIC100) in tablet or capsule form was found to be 2.5 µg/mL. Interestingly, *E. purpurea* tea brewed at 80 °C showed a MIC100 of 2.2 µg/mL, indicating that the tea preparation of *E. purpurea* may offer superior antiviral activity compared to capsules or tablets. Among several extracts of *E. pallida* var. *angustifolia* roots (55% ethanol extract at 40 °C, water extracts at 40 °C and 80 °C, 70% ethanol extract at 40 °C, hexane, and ethyl acetate extracts), only the 55% ethanol extract at 40 °C and the ethyl acetate extract displayed anti-influenza effects [127]. Notably, the ethyl acetate extract of *E. pallida* var. *angustifolia* exhibited the strongest antiviral activity against influenza, with a MIC100 of 33.5 µg/mL, significantly outperforming the ethanol extract, which had a MIC100 of 348 µg/mL [128].

**Figure 5:**
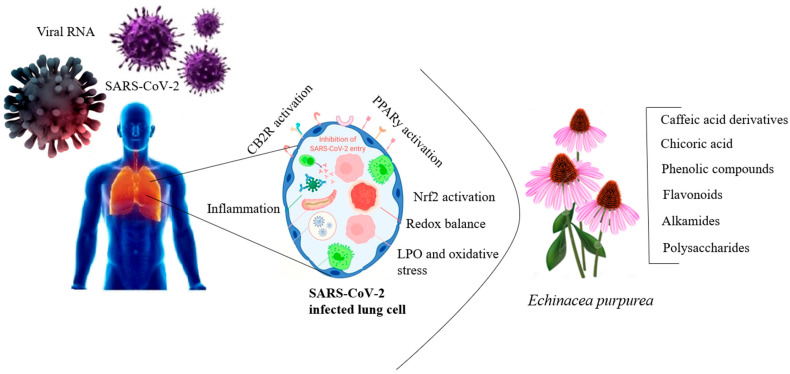
Antiviral activity of echinacea extract against SARS-CoV-2 virus (Figure drawn by F. Ahmadi).

Research has been conducted on the antiviral effects of echinacea extract against the SARS-CoV-2 virus [129]. SARS-CoV-2 enters host cells by interacting with ACE2 receptors, and the virus’s pathogen-associated molecular patterns (PAMPs) activate innate immune cells, including antiviral effectors like CD8+ T cells, neutrophils, monocytes, and macrophages, in response to the infection [130]. Innate immune cells, equipped with pattern recognition receptors (PRRs) such as Toll-like receptors (TLRs), retinoic acid-inducible gene I (RIG-I)-like receptors (RLRs), and nucleotide-binding oligomerization domain (NOD)-like receptors (NLRs), detect PAMPs and trigger an immune response against the invading pathogen [131]. The interaction between PRRs and PAMPs stimulates phagocytosis and leads to the production of proinflammatory cytokines, including type I interferons (IFNα/β), type II interferons (IFN-γ), and chemokines like interferon gamma-induced protein 10 and monocyte chemoattractant protein-1, generating an antiviral response [132]. The immunomodulatory properties of echinacea induce an antiviral response by influencing the activity of PRRs, PAMPs, and damage-associated molecular patterns (DAMPs). Echinacea has demonstrated efficacy against rhinoviruses [133], influenza [134], respiratory syncytial virus (RSV) [135], herpes virus [136], adenoviruses [137], and coronaviruses [138]. Additionally, chicoric acid, a compound found in echinacea, has shown potent antiviral activity against herpes simplex, influenza, enterovirus, hepatitis B, vaccinia virus, and vesicular stomatitis virus-Ebola [139]. In HIV-1, chicoric acid and its derivatives inhibit the enzyme integrase, which is responsible for integrating viral DNA into the host genome, a crucial step in viral replication [140] (Figure 5). Treatment with echinacea has also been shown to preserve the activity of natural killer cells and monocytes, providing nonspecific immunity, and helping to eliminate virus-infected cells [141].

In other in vitro studies, the antiviral effects of an aqueous solution of *E. purpurea* herb were tested against aciclovir-susceptible and aciclovir-resistant strains of HSV-1 and HSV-2 [142]. For aciclovir-susceptible strains of HSV-1 and HSV-2, the median effective dose (ED50) values for the echinacea preparation were 1:100 (ranging from 1:25 to 1:400) and 1:200 (ranging from 1:50 to 1:1600), respectively. Similarly, for aciclovir-resistant HSV-1 and HSV-2, the median ED50 values were 1:100 (ranging from 1:50 to 1:400) and 1:200 (ranging from 1:50 to 1:3200), respectively [143]. In light-activated antiviral activity studies, an n-hexane extract of *E. purpurea* root, an ethanolic extract of *E. pallida* var. *sanguinea* herb, and the isolated compound chicoric acid were the most potent inhibitors of HSV-1, with minimum inhibitory concentrations of 0.12, 0.026, and 0.045 mg/mL, respectively [144]. Additional in-vitro studies using mouse fibroblasts demonstrated that pre-incubation with *E. purpurea* herb juice and methanolic and aqueous extracts of *E. purpurea* root provided 24-h resistance to infections caused by influenza A2, herpes, and the vesicular stomatitis virus [145].

## 7. Mechanism of Antiviral Activity of Polyphenols

### 7.1. Inhibition of Virus Entry

Echinacea demonstrates antiviral activity through several key mechanisms, starting with the inhibition of viral entry into host cells [146], a vital step in halting the infection process. Compounds such as caffeic acid derivatives (including chicoric acid and echinacoside) and flavonoids (like quercetin and kaempferol) in echinacea can bind to viral surface proteins or host cell receptors [147]. This interaction effectively blocks the virus from attaching to host cells, which is an essential initial phase in the viral replication cycle [148]. Additionally, these compounds can interfere with the viral envelope or disrupt the host cell membrane, particularly in enveloped viruses, preventing the fusion process needed for the virus to release its genetic material into the host cell. By obstructing both attachment and fusion, echinacea compounds substantially reduce the likelihood of viral entry and subsequent infection.

### 7.2. Inhibition of Viral Replication

Another key mechanism by which echinacea exerts its antiviral effects is through the inhibition of viral replication [149]. Once a virus successfully enters a host cell, it must replicate its genetic material to produce new viral particles. Polyphenols found in echinacea, such as chicoric acid and rosmarinic acid, can disrupt this process by inhibiting viral RNA and DNA polymerases—enzymes that are essential for replicating viral genetic material [150]. By blocking these polymerases, these compounds effectively stop the virus from replicating its genome, thereby preventing the production of new viral particles. Moreover, echinacea extracts can interfere with viral protein synthesis by binding to viral mRNA or the host’s ribosomes. This disruption of protein translation is crucial, as it hinders the assembly of new virions, ultimately reducing the viral load within infected cells [151].

### 7.3. Modulation of Host Immune Response

Echinacea also influences the host’s immune response, which is crucial for its antiviral effectiveness. The plant’s compounds can boost the activity of immune cells like macrophages, dendritic cells, and natural killer cells, all of which play vital roles in identifying and eliminating virus-infected cells [152]. Alkamides, caffeic acid derivatives, and polysaccharides found in echinacea activate these immune cells, enhancing their capacity to detect and destroy infected cells early in the infection process [153]. Moreover, echinacea compounds stimulate the production of cytokines, particularly interferons, which are key signaling molecules involved in antiviral defense. Interferons induce an antiviral state in nearby cells and strengthen the adaptive immune system, making it more effective in fighting viral infections [154].

### 7.4. Antioxidant and Anti-Inflammatory Effects

Echinacea’s antiviral effects are also supported by its antioxidant and anti-inflammatory properties. Viral infections often trigger oxidative stress, which can result in damage to host cells and tissues [155]. Polyphenols, such as chicoric acid and rosmarinic acid, possess potent antioxidant capabilities, helping to neutralize reactive oxygen species and mitigate oxidative stress, thereby protecting host cells from harm [156]. This protective action not only aids the immune system but also strengthens the body’s capacity to fend off viral infections [156]. Beyond their antioxidant properties, echinacea compounds have notable anti-inflammatory effects, which are especially beneficial during viral infections. Excessive inflammation can be harmful and lead to tissue damage. Polyphenols like flavonoids and caffeic acid derivatives can suppress the production of pro-inflammatory cytokines, such as TNF-alpha and IL-6, along with enzymes like cyclooxygenase-2, thus helping to regulate the inflammatory response [157]. By controlling inflammation, echinacea promotes a balanced immune reaction, enabling viral clearance while minimizing tissue damage [157].

### 7.5. Inhibition of Virus Release

Echinacea can inhibit the release of viruses from infected cells, a critical step in the viral life cycle that allows the infection to propagate [158]. Some studies indicate that echinacea compounds disrupt the later stages of viral replication, particularly the assembly and release of new viral particles [159]. This interference may result from interactions between polyphenols and viral proteins or host cell membranes, preventing the formation of mature virions or hindering their ability to bud off from the host cell surface [160]. By blocking these processes, echinacea helps to limit viral spread within the host, enhancing its overall antiviral effectiveness.

## 8. Immunomodulatory Activity

The term “immunomodulatory” is increasingly seen as more appropriate than “immunostimulatory” to describe echinacea’s immunological effects, although the latter remains widely used in earlier scientific discussions on the plant [161]. It has been suggested that broadly stimulating the highly complex immune system may not always be beneficial, as some immune responses can be detrimental [162]. Numerous studies, both in vitro and in vivo, have explored the immunological effects of various echinacea preparations, covering different species, plant parts, and extraction methods. Collectively, the findings suggest that echinacea preparations do impact certain immune functions, though there is no consensus on which specific preparations have the strongest effects [163]. Enhanced macrophage function has been observed in response to different echinacea formulations, as demonstrated by in vitro and in vivo studies using methods like the carbon-clearance test and cytokine measurement to assess macrophage activity [164]. In vitro studies on human macrophages found that fresh-pressed juice and dried juice from the aerial parts of *E. purpurea* stimulated the production of cytokines such as interleukin (IL)-1, IL-10, and tumor necrosis factor (TNF-α) [165].

Other research has reported that purified polysaccharides from *E. purpurea* triggered macrophages to produce IL-1 [166], while an arabinogalactan polysaccharide, isolated from *E. purpurea* plant cell cultures, was found to induce the production of TNF-α and interferon-2 in murine macrophages [167]. Polysaccharides from *E. purpurea* plant cell cultures have also been previously shown to exhibit immunological activity in vitro [168]. In another series of in vitro experiments, *E. purpurea* promoted macrophage activation, as indicated by TNF-α production, following simulated digestion (incubation with gastric fluid) to replicate the potential effects of oral consumption [169]. Further studies demonstrated that *E. purpurea* dry root powder (containing 1.5% total polyphenols, calculated as chlorogenic acid) enhanced the resistance of splenic lymphocytes to apoptosis. This effect was seen in splenic lymphocytes from mice that received the echinacea preparation orally at doses of 30 or 100 mg/kg daily for 14 days [170].

In an in vitro study, peripheral blood mononuclear cells from healthy individuals, as well as from patients with chronic fatigue syndrome, were exposed to increasing concentrations of *E. purpurea* extracts, which resulted in enhanced natural killer cell function [171]. Similarly, in vivo studies have shown that oral administration of *E. purpurea* root extract increases natural killer cell numbers in normal [172], leukemic [173], and aging mice [174]. A subsequent in-vivo study using a randomized, double-blind design examined the effects of an echinacea supplement (Nature’s Resource; CVS Pharmacy, USA; containing 1.05 g of echinacea aerial parts and 10.5 mg of chicoric acid) in 16 aging male rats [175]. The animals received 50 mg/kg body weight of echinacea (potentially *E. purpurea*), equivalent to 0.5 mg/kg of chicoric acid, or a placebo administered in peanut butter daily for 8 weeks. During the first two weeks, echinacea-treated rats showed significantly higher mean circulating white blood cell counts compared to the control group (*p* < 0.05) [176]. IL-2 concentrations were significantly higher in the echinacea group compared to the control for the last five weeks of the study (P<0.05). Additionally, differential white cell counts changed markedly over the 8 weeks: lymphocyte and monocyte levels increased, while neutrophil and eosinophil levels decreased in the echinacea group compared to placebo [177].

No changes in the phagocytic activity of circulating leukocytes, measured by their ability to ingest latex particles, were observed in either group. Other in-vivo studies in rats demonstrated that water-ethanol extracts of *E. purpurea* roots and aerial parts, containing chicoric acid, polysaccharides, and alkamides, increased macrophage phagocytic activity, with greater concentrations of these components leading to enhanced activity [178]. Moreover, spleen macrophages from echinacea-treated rats displayed increased nitric oxide release when stimulated with lipopolysaccharide. In a similar experiment, alkamides were shown to stimulate alveolar macrophage function in healthy rats [179].

**Figure 6:**
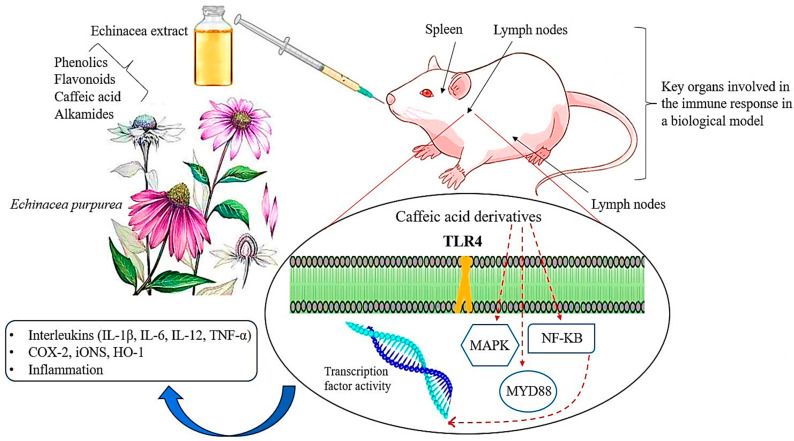
A schematic representation of the main molecular pathways linked to inflammatory and immunomodulatory activities modulated by echinacea. The solid red line indicates the pathway’s activation, whereas the truncated red line indicates inhibition of the pathway. TLR-4: Toll-like Receptor-4; MyD88: Myeloid Differentiation Primary Response 88; NF-KB: Nuclear Factor kappa B; MAPK: Mitogen-Activated Protein Kinase; COX-2: cyclooxygenase-2; iNOS: inducible Nitric Oxide Synthase; HO-1: Heme Oxygenase-1; IL: Interleukin; TNF: Tumor Necrosis Factor (Figure drawn by F. Ahmadi).

A proprietary preparation combining *E. purpurea* root extract and licorice root extract [180] has been found to stimulate phagocytosis both in vitro and in vivo, as evidenced by the carbon-clearance test following oral administration in mice [181] (Figure 6). This combination demonstrated a more potent immunostimulatory effect compared to either extract used independently. Another blend, consisting of aqueous ethanol extracts from *E. purpurea* and *E. pallida* roots, *Baptisia tinctoria* root, and *Thuja occidentalis* herb, was administered orally to mice through their diet or drinking water for 7 days, resulting in an enhanced antibody response to sheep red blood cells [182]. However, despite the substantial research supporting the immunostimulatory properties of echinacea preparations, some recent studies have reported no such effects [183]. For example, no evidence of natural killer cell activity or antibody production was observed in studies where rats were fed various echinacea formulations, including alcoholic extracts of *E. purpurea* root and alcoholic extracts of the roots of *E. angustifolia*, *E. purpurea*, and *E. pallida*, in their diet [184].

## 9. Conclusions and Future Perspective on Echinacea Research

Echinacea’s phenolic and flavonoid compounds, including caffeic acid derivatives and alkamides, are known for their antimicrobial properties [185]. Studies have shown that the phytochemicals in *E. purpurea* significantly reduce inflammatory swelling in mice and rats and lower blood levels of cytokines such as IL-2, IL-6, and TNF-α [186]. These effects are similarly observed with the administration of alkylamides in animal models. With continued research and the establishment of standardized extraction methods, echinacea extract has the potential to become a successful product line in the echinacea industry [187]. Currently, echinacea is predominantly marketed in the US as an immune-boosting supplement [188]. Echinacea alkylamides have been shown to disrupt bacterial cell walls and membranes, with one structural class, the diynoic alkylamides, demonstrating the greatest cell wall disruption [189,190]. The natural variation in phytochemical profiles across echinacea species provides the opportunity to selectively propagate cultivars with unique metabolic profiles for specific purposes, such as producing antimicrobial compounds. Alternatively, echinacea cultivars could be bred to increase the production of caffeic acid derivatives in the flowers, which could be used in dietary supplements. Roots and flowers rich in alkylamides and chicoric acid could be used to produce antibacterial face washes, shampoos, or creams. Although echinacea, like other members of the Asteraceae family, contains some phototoxic polyacetylene compounds, these are unstable and can be easily deactivated by minimal processing [187]. Additionally, the leaves of echinacea, which are rich in vitamin C and phenolic compounds, could be marketed as natural health products. However, the full potential of echinacea’s diverse applications remains largely untapped. The development of new biotechnologies presents significant opportunities for improving echinacea-based products [190,191]. Moving forward, it is important to weigh the benefits and drawbacks of different approaches, such as improving yields, optimizing phytochemical profiles, enhancing propagation efficiency, managing costs, addressing public perception, and ensuring scalability. A combination of tissue culture, chemical treatments, and traditional field cultivation will likely be used to achieve higher production standards and improved phytochemical quality. These advancements will allow the industry to expand the range of echinacea products and meet the growing market demand.

## Figures and Tables

**Table 1:** Hydrocarbons from Rhizomes ^a^ of echinacea species [46].

Species	Hydrocarbon
*E. angustifolia*	Dodeca-2,4-dien-1yl isovalerate (Z)-Pentadeca-1,8 diene Pentadec-1-ene
*E. pallida*	(Z)-Pentadec-8-en-2-one
	(Z)-Pentadeca-1,8-diene Pentadec-1-ene
	(8Z,11Z)-Pentadeca-8,11-dien-2-one
	(8Z,13Z)-Pentadeca-8,13-dien-11-yn-2-one
	(Z)-Tetradeca-8-diene-11,13-diyn-2-one
	(E)-10-Hydroxy -4,10-dimethydodeca-4, 11-dien-2-one (echinolone)
	(Z)-Pentadeca-8-ene-11,13-diyn-2-one
	(8Z,11Z,13E)-Pentadeca-8,11,13-trien-2-one
	(8Z,11E,13Z)-Pentadeca-8,11-trien-2-one
*E. purpurea*	Dodeca-2,4-dien-1-yl isovalerate

^a^ Referred to as roots in most references.

## Data Availability

Data are available upon request.
